# A Multi-Physics Modeling Framework for Optimizing Spreading and Sintering Parameters in Powder Bed Fusion

**DOI:** 10.3390/polym18131663

**Published:** 2026-07-04

**Authors:** Jiang Li, Fulun Peng, Jianzhao Zhao, Xinliang Chai, Junjie Fu, Shaoying Li, Xujiang Chao

**Affiliations:** 1 Xi’an Institute of Applied Optics, Reconnaissance Vehicle Research and Development Center, Xi’an 710065, China; pcoolfy@163.com (F.P.); zjz2046163@163.com (J.Z.); chaixl@mail.nwpu.edu.cn (X.C.); fujunjie@stu.xjtu.edu.cn (J.F.); 2 Xi’an Modern Control Technology Research Institute, Xi’an 710061, China; lishaoying@mail.nwpu.edu.cn; 3 School of Mechanical Engineering, Northwestern Polytechnical University, Xi’an 710072, China

**Keywords:** powder bed fusion, process window optimization, PEEK/carbon fiber composites, discrete element method and finite element method, powder spreading and laser sintering

## Abstract

Powder Bed Fusion-Laser Beam/Polymer (PBF-LB/P) is a key additive manufacturing technology widely used in aerospace, but its process parameters are difficult to optimize for thermoplastic composites due to poor powder flowability and unstable melting regions. To address this challenge, this paper develops discrete element and finite element models to systematically determine the PBF process window for both powder spreading and sintering stages, with verified reliability. In the spreading stage, the powder layer performance is evaluated through surface profile, density, and uniformity. The effects of reinforcement phase, spreading speed, and layer thickness are analyzed, establishing reasonable spreading parameter windows. It is found that the optimal layer thickness for PEEK powder is determined to be 0.13 mm, while that for PEEK/CF composite powder is 0.12 mm. At the optimal layer thickness, the powder bed exhibits desirable properties, which minimize its adverse influence on the sintering process and serve as a prerequisite for subsequently establishing the sintering process window. For the sintering stage, sufficient sintering constraint criteria are established, and a systematic determination method is proposed. By analyzing microscopic sintering mechanisms and characterizing the effects of laser power, scanning speed, and hatching space on melt pool dimensions and temperature, a reasonable sintering process window can be efficiently determined. It is found that within the process window, the PEEK specimens achieved a maximum relative density of 99.31% and exhibited a tensile strength 13.1% higher than that of specimens processed outside the window, demonstrating a clear superiority.

## 1. Introduction

### 1.1. Research Background

Powder Bed Fusion-Laser Beam/Polymer (PBF-LB/P) is an additive manufacturing technique that fabricates components layer by layer. A laser selectively sinters powder bed areas based on part cross-sectional data. The apparatus includes a powder delivery system, fabrication chamber, roller, laser source, and control system. The process begins with a CAD model, which is sliced into layers of predefined thickness. Key parameters—preheating temperature, laser power, scanning speed, and layer thickness—are established. The powder feed piston elevates material, and a counter-rotating roller spreads a uniform layer onto the build platform. The laser scans and sinters the powder according to preset parameters. After each layer, the platform is lowered, and a fresh powder layer is spread. This cycle repeats until fabrication is complete. PBF offers several advantages over other additive manufacturing technologies. It accommodates a wide range of materials; any powder whose viscosity decreases sufficiently upon laser absorption can be processed. Material utilization is high, as unsintered powder can be recycled. Additionally, dedicated support structures are typically unnecessary, as the surrounding powder bed provides inherent support for overhanging features and internal cavities [1,2,3].

**Figure 1:**
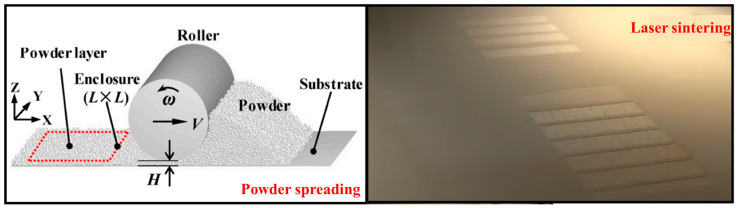
Schematic diagram of the powder spreading and sintering stages during the PBF process (original).

The operational process of PBF comprises two distinct stages: powder spreading and laser sintering (Figure 1). To achieve superior component performance, process parameters for each stage must be optimized. During powder spreading, powder flowability significantly influences deposited layer quality. Key parameters include roller translational speed and layer thickness. Layer quality is evaluated by surface flatness, density, and density uniformity. High surface flatness facilitates subsequent sintering and next-layer deposition. High and uniform density reduces porosity and minimizes light scattering, thereby enhancing energy absorption during laser irradiation [4,5,6]. Additionally, roller speed substantially affects process efficiency; thus, maximizing speed while maintaining layer quality is crucial for productivity. For laser sintering, key parameters include laser power, scanning speed, beam offset, and hatching space. For a given layer thickness, insufficient laser energy density may prevent melt pool penetration through the full layer depth, leading to inter-layer delamination and compromising mechanical properties or causing build failure [7,8]. Conversely, excessive energy density causes melt pool penetration beyond the layer thickness, potentially remelting previously solidified material and introducing defects such as over-melting, which also adversely affects mechanical performance [9,10]. Therefore, well-calibrated processing parameters are essential to avoid insufficient sintering or overheating, ensuring complete and proper material consolidation.

In summary, PBF is a complex process involving sequential sub-processes and multi-physics fields. Therefore, it is essential to investigate the underlying microscopic mechanisms of each stage and identify optimal process parameter combinations to provide effective and reliable guidance for practical PBF operations.

Compared with metallic and ceramic materials, polymers offer distinct advantages for laser-based powder bed fusion (PBF-LB/P)—the standard terminology defined in ISO/ASTM 52900 [11] for the laser-based polymer powder bed process, historically also known as selective laser sintering (SLS), including lower sintering temperatures, reduced laser power requirements, and higher dimensional accuracy [12]. The diversity of polymeric materials and associated modification technologies has significantly expanded their application scope. Commonly used semi-crystalline polymers include Polyether-ether-ketone (PEEK) and PA12. PEEK is renowned for its excellent strength, stiffness, high thermal resistance, self-lubricating properties, flame retardancy, and electrical insulation, enabling broad applications in weaponry, aerospace, aviation, and automotive manufacturing [13,14,15]. However, several challenges persist in PBF-LB/P processing of PEEK. Its melting point exceeds 300 °C, imposing high demands on equipment [16]. Its poor powder flowability complicates the spreading process, potentially affecting subsequent sintering. Additionally, identifying appropriate sintering parameters is difficult, as either excessive or insufficient laser energy can cause build failure [13,14]. Therefore, this study focuses on simulating the PBF-LB/P process for thermoplastic polymers, selecting PEEK as a representative material to accurately predict its viable processing window.

### 1.2. Research Status

#### 1.2.1. Research on Powder Spreading Process

PBF-fabricated parts made from thermoplastic polymers and their composites exhibit favorable mechanical properties, including high specific strength and modulus [17,18]. However, inappropriate processing parameters can lead to defect retention, often causing brittle failure. The origins of these internal defects trace back to the powder spreading stage [19,20]. Powder layer quality significantly influences subsequent heat transfer, sintering efficiency, and porosity formation in the final part [21,22]. Therefore, it is essential to investigate the microscopic mechanisms of powder spreading and optimize associated process parameters based on detailed characterization of powder material properties—a prerequisite for achieving high performance in fabricated components.

To systematically investigate the powder spreading process, two interconnected tasks must be addressed. The first is the calibration of the Discrete Element Model (DEM) parameters, as the accuracy of DEM simulations relies heavily on the reliable assignment of material-specific input parameters (e.g., particle–particle and particle–wall friction coefficients, restitution coefficients, and van der Waals forces). Without proper calibration, the subsequent predictions of powder flowability and layer quality would lack credibility [23]. The second task is a comprehensive simulation study of the powder spreading mechanism, which aims to elucidate how particle-level interactions and spreading parameters collectively determine the deposited layer quality, and to identify the optimal spreading conditions for thermoplastic polymers and their composites.

(1) Parameter Calibration of the Discrete Element Model (DEM)

The DEM model used in this study is adopted from our previous work [24], where the theoretical formulation, parameter calibration, and experimental validation were comprehensively detailed. That prior work established the Hertz–Mindlin with JKR contact model, the ‘PB-BBD-GA’ parameter-design method, and the particle shape representation strategy (spherical particles with rolling friction for PEEK and a multi-sphere clump method for CF). The present study builds upon this established model by applying it to systematically investigate the spreading behavior of PEEK and PEEK/CF, and more importantly, by sequentially integrating the DEM-derived optimal spreading parameters into FEM thermal simulations to establish a full-process SLS process window.

(2) Simulation Study of the Powder Spreading Mechanism

During powder spreading, the discontinuous and stochastic nature of particles leads to complex interaction and motion mechanisms. Powder flowability and spreading parameters jointly determine the deposited layer quality [25,26,27]. Based on DEM simulations, Chen et al. [28] found that reducing inter-particle friction enhances flowability, yielding denser and more uniform layers. Haeri et al. [25] showed that a super-elliptical spreader profile reduces porosity and surface profile. Parteli et al. [29] reported that higher spreading speeds increase surface profile. Ma et al. [30] demonstrated that enhanced van der Waals forces reduce flowability and degrade layer quality. Nan et al. [31] observed that mass flow rate increases with both spreading speed and layer height. Other studies have investigated microscopic mechanisms. Yao et al. [32] focused on the interface between the fabricated region and fresh powder layer, finding a significant increase in normal contact force chains that reduces packing density. Chen et al. [33] identified three deposition mechanisms-adhesion effect, wall effect, and percolation effect that dominate powder layer packing density.

However, current research on the PBF powder spreading process faces several challenges. First, significant adhesive forces between thermoplastic polymer particles exert a pronounced influence on spreading behavior and powder layer properties. Thus, the spreading mechanisms and optimal process windows for thermoplastic polymers and their composites require urgent investigation. Second, with the introduction of a reinforcing phase, inter-particle forces become more complex, encompassing interactions among reinforcing phase particles, between reinforcing phase and polymer particles, and among polymer particles themselves. The influence of these concurrent forces on powder flowability and layer properties is complex and requires further study. Third, for polymers and their composites, the combined effects of inter-particle forces and spreading parameters on layer characteristics are intricate. Therefore, well-defined metrics are needed to evaluate the impact of material properties and spreading parameters, enabling the determination of a reasonable and effective powder spreading process window. These identified challenges motivate the DEM simulations and parametric analyses presented in the subsequent sections of this study.

#### 1.2.2. Research on Sintering Process

Simulation of the powder spreading phase lays the foundation for achieving excellent properties in PBF-formed parts. For the subsequent sintering process, a reasonable sintering process window must be explored. A thermo-mechanical model based on the Finite Element Method (FEM) is commonly used to simulate the PBF process and predict part deformation. Zeng et al. [34] reviewed heat transfer in PBF and predicted temperature field distribution during laser scanning. Parry et al. [35] employed a simplified thermo-mechanical model to predict residual stresses from temperature gradients. Li et al. [36] incorporated temperature-dependent material properties and found that residual stresses are larger along the scanning direction. Tan et al. [37] developed a model including the “powder-liquid-solid” phase transition and found that it increases compressive stress while decreasing tensile stress during scanning. Inappropriate sintering parameters can affect surface properties, mechanical strength, forming efficiency, and dimensional accuracy [38,39,40]. Yuan et al. [41] evaluated laser-polymer powder interaction, considering chamber conditions, laser parameters, temperature-dependent material properties, and phase transition in their numerical model, establishing quantitative relationships between process parameters and multiple optimization objectives.

However, current research on sintering process parameters exhibits the following shortcomings. First, prevailing research methods often rely on a trial-and-error approach with controlled variables, seeking reasonable parameter combinations by adjusting multiple parameters. Given the multitude of sintering parameters—including laser power, scanning speed, hatching space, and beam offset—this approach leads to low research efficiency and questionable reliability. Therefore, it is essential to explore the microscopic mechanism of sufficient sintering and clarify how process parameters influence the sintering process, enabling a more systematic and efficient determination of the sintering process window. Second, most current studies focus on single-objective optimization, often yielding a specific set of process parameters. However, parameters at different locations within the sintering region can be further optimized to meet practical requirements. For example, contour process parameters significantly impact the surface properties of formed parts. Optimizing contour and interior parameters separately can ensure excellent surface properties and mechanical performance. Nevertheless, the influence mechanism of contour parameters on surface properties remains unclear, necessitating exploration of the key factors by which the contour region affects surface quality.

Therefore, addressing the aforementioned challenging issues, this paper presents innovative research in the mechanisms of powder spreading and sintering processes. At the level of powder spreading process research, simulation studies are conducted on the powder spreading process for polymers and their composites in PBF. This enables the matching of powder spreading process parameters with powder flowability, aiming to minimize the negative impact of powder layer properties on the subsequent sintering process. Powder layer performance is evaluated based on surface profile, density magnitude, and density distribution uniformity. A method for partitioning and quantifying the powder layer is proposed. This approach allows for a more accurate comparison of powder spreading effects under different processes and improves the accuracy of predicting the powder spreading process window. The effects of spreading speed and layer thickness are analyzed, clarifying the influence of the reinforcement phase on the powder spreading mechanism and powder layer performance. PEEK and PEEK/carbon fiber (CF) powders, characterized by poor flowability and significant spreading difficulty, are selected as the most representative research subjects. Their reasonable powder spreading process window is clarified, and the optimal powder layer thickness for PEEK is determined, thereby establishing key conditions for subsequent sintering process parameters.

At the level of sintering process research, this paper established constraint criteria for sufficient sintering in PBF, proposed a systematic method for determining the sintering process window, and separately determined the process parameters for the contour and interior of the sintering region. The microscopic mechanism of sufficient sintering is analyzed, reflecting the impact of different sintering process parameters on the sintering process through melt pool dimensions and temperature. Using an FEM model, the dimensions and temperature information of the sintering melt pool are calculated. Taking the optimal layer thickness as an input condition, a reasonable sintering process window is determined based on common polymers and their composites, and its reliability is fully verified through experiments.

## 2. Material and Method

### 2.1. Material

#### 2.1.1. Material Parameters for the DEM Model

**Figure 2:**
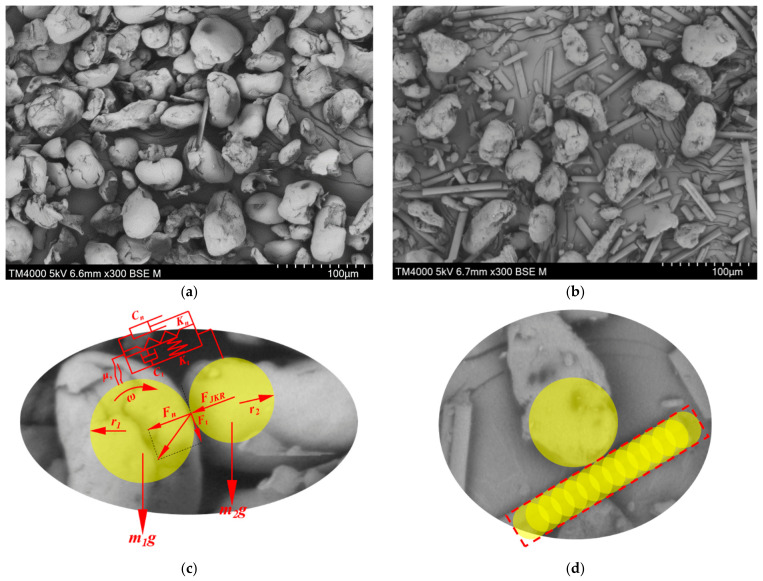
SEM images showing the morphology of powder particles: (**a**) PEEK; (**b**) PEEK/CF_30wt %; (**c**) Simplified schematic of particle contact; (**d**) Schematic illustration of the multi-sphere clump method used to approximate the elongated morphology of CF particles in the DEM model (reproduced from our previous work [24]).

This work selects the materials of PEEK (ZYPEEK_330PF, Jilin Joinature Polymer Co., Ltd., Changchun, China) and PEEK/CF (CF from NanJing WeiDa Composite Material Co. Ltd., Nanjing, China) powders, which possess the features of poor powder fluidity and great difficulty in the powder deposition process, as the representative research object. These are designated as PEEK, PEEK/CF_30wt %, and PEEK/CF_50wt %, respectively. For the latter two, this denotes that the mass fraction of CF within the PEEK/CF composite powder is 30% and 50%, respectively. The material parameters in the powder spreading simulation model include particle morphology, particle size distribution, coefficient of static friction, coefficient of rolling friction, coefficient of restitution, and surface energy. Among these, the particle size distribution is a crucial input, as it significantly influences the computational efficiency of the model. Figure 2 presents the SEM (Scanning Electron Microscope, TM4000PLUS, Hitachi High-Tech Corporation, Tokyo, Japan) test results for PEEK and PEEK/CF_30wt %. It is observed that nearly all PEEK powder particles are ellipsoidal, with the majority of PEEK particles having a size ranging from 30 to 60 μm. In the subsequently described DEM model adopted from our prior work, PEEK particles are represented as spheres, while CF particles are modeled using a multi-sphere (clump) method to approximate their elongated morphology (see Figure 2d for a schematic illustration of the CF clump representation). Although PEEK particles are irregular in reality (as shown in the SEM images in Figure 2), the spherical simplification is compensated by assigning higher rolling friction coefficients to effectively replicate the rotational resistance of non-spherical particles. The rolling friction coefficients were systematically calibrated using the ‘PB-BBD-GA’ method developed in our prior work [24].

**Figure 3:**
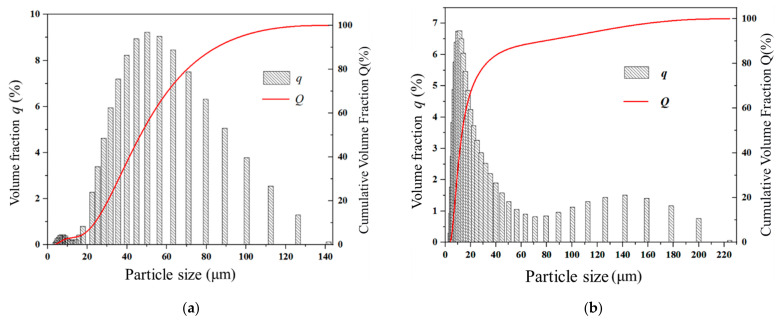
Particle size distribution of powder materials: (**a**) PEEK; (**b**) CF (reproduced from our previous work [24]).

A laser particle size analyzer can accurately measure the particle size distribution information of the powder. The particle size distribution results for PEEK and CF are shown in Figure 3 and Table 1. It can be found that the vast majority of PEEK particles have a size ranging from 30 to 60 μm.

The detailed material parameters of particle–particle and particle–wall are shown in Table 2 below. The coefficient of static friction between PEEK materials is measured through incline tests and is determined to be 0.4689 ± 0.0064. The coefficient of restitution is obtained via FEM simulation, yielding a value of 0.2746. The coefficient of rolling friction and the surface energy are acquired using the “PB-BBD-GA” parameter design method, with values of 0.1305 and 0.005, respectively (detailed parameter calibration methods are provided in the preceding paper). It should be noted that the wall material in the DEM simulations is assumed to be glass, consistent with the calibration experiments in our prior work [24]. This choice ensures consistency with the calibrated parameters and maintains the predictive validity of the model. Although the actual roller material in the SLS equipment may differ, we emphasize that particle–wall contact parameters have a significantly smaller influence on powder layer quality compared with particle–particle interactions, as demonstrated in our prior sensitivity analysis. Moreover, the substrate–particle interactions only affect the first deposited layer, since subsequent layers are spread onto previously deposited powder layers rather than directly onto the substrate. Therefore, the use of glass wall parameters is considered a reasonable simplification for the parametric comparative analysis conducted in this study.

#### 2.1.2. Thermal and Mechanical Material Properties

The fundamental thermodynamic properties of PEEK are presented in Table 3 [42].

The material properties and model parameters related to PEEK are described as follows.

**Figure 4:**
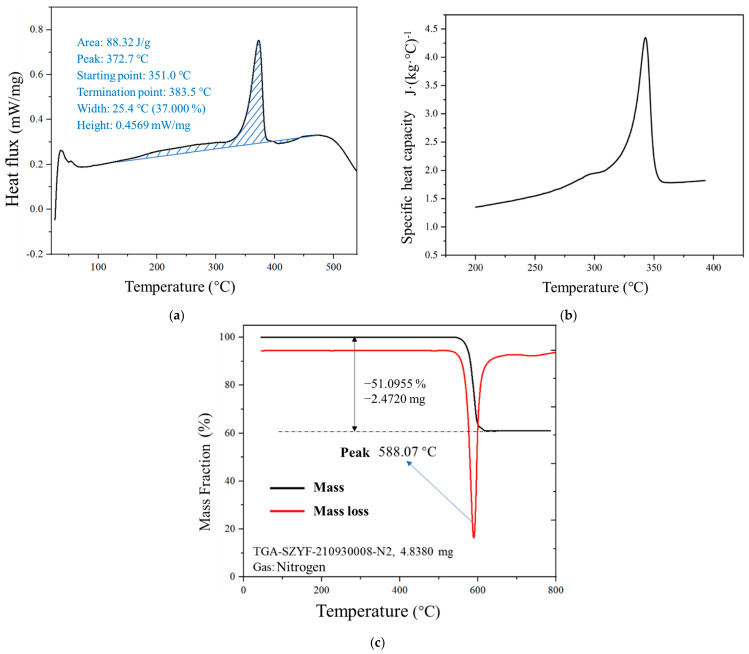
Material Properties of PEEK: (**a**) DSC results; (**b**) Specific heat capacity; (**c**) TGA thermogravimetric curve.

(1) Using a Differential Scanning Calorimeter (DSC), a certain amount of PEEK powder is heated from room temperature to above its melting point at a heating rate of 10 °C/min under a nitrogen atmosphere. The heat flow-temperature information during the heating process is measured to obtain the onset and endpoint temperatures of the melting region, as shown in Figure 4a. The preheating temperature of the powder bed should be set slightly below the onset temperature of the melting region, and can be set to 330 °C. Additionally, Figure 4b illustrates the relationship between the specific heat capacity of the PEEK material and temperature, reflecting the nonlinear variation in the material with temperature during the sintering process [43].

(2) Thermogravimetric analysis (TGA) is performed using a thermogravimetric analyzer (STA 449F3, NETZSCH, Selb, Germany) to measure the decomposition temperature of the PEEK material. Approximately 5 mg of the PEEK powder sample is heated from room temperature to 800 °C at a rate of 10 °C/min under a nitrogen environment, yielding the material decomposition curve, as shown in Figure 4c. The thermal decomposition temperature of the PEEK material is approximately 588.07 °C. Therefore, the suitable sintering region (SSR) for PEEK is within the range of 383.5 °C to 588.07 °C.

(3) Based on the preceding studies, when the powder layer thickness is set to its optimal value, the density of the powder layer approaches its tap density. This density corresponds to the initial powder bed density employed in the FEM model. The experimentally measured tap density of the PEEK powder is 0.4157 g/cm^3^. By comparing this with its solid-phase density of 1.3 g/cm^3^, the porosity can be calculated as 1 − (0.4157/1.3) ≈ 0.68 [24]. Consequently, when inputting parameters into the computational model, the influence of porosity on relevant material properties, such as density and thermal conductivity, must be considered. This necessitates a distinction between the material properties in its powdered state and its solid/liquid state.

(4) The attenuation coefficient and the laser energy absorptivity in the subsequently described heat source equation are referenced from the testing methodology and measurement results reported by Professor Xiaoyong Tian’s research group at Xi’an Jiaotong University [43,44].

In the subsequent FEM model, the powder layer is distinguished from the consolidated material through porosity-dependent material properties. The initial porosity of the powder bed is calculated from the measured bulk density (0.4157 g/cm^3^) and solid density (1.3 g/cm^3^) of PEEK, giving a porosity of approximately 68%. For temperatures below the melting onset (351 °C), the effective density and thermal conductivity are scaled by the porosity to represent the powder state. For temperatures above the melting point (383 °C), the material is treated as fully dense. A linear interpolation is applied in the mushy zone between these two temperatures. All temperature-dependent material properties are input as tabulated functions in Abaqus, with the porosity correction applied to the powder state as described. All material properties, including density and thermal conductivity, are input as temperature-dependent functions in Abaqus, with the porosity correction applied to the powder state as described above. The absorptivity of the powder bed is also adjusted to account for the porous structure, following the effective medium approach. Moreover, it is acknowledged that latent heat and crystallinity-related effects are not explicitly included in the current thermal model. These effects are expected to have a secondary influence on the melt pool dimensions and temperature fields compared with the porosity-driven property changes, and their inclusion would refine rather than fundamentally alter the predicted process window. This has been noted as a direction for future model refinement [45].

### 2.2. Method

The DEM and FEM models are employed in a sequential manner in this study. The DEM model is first used to identify the optimal spreading parameters (primarily the layer thickness) that yield a powder bed with optimized density and uniformity. The optimal layer thickness is then transferred to the FEM model as the input condition for thermal simulations. The FEM model assumes a homogeneous powder bed, which is justified by the DEM results showing that under the optimal spreading conditions, the powder bed is sufficiently uniform and dense. This sequential approach ensures that the FEM simulations are performed under the most favorable powder bed conditions identified by the DEM analysis. The DEM spreading analysis covers both PEEK and PEEK/CF powders to investigate the effect of CF reinforcement on spreading behavior. The sintering process window and experimental validation are established for PEEK material. The sequential DEM-FEM framework is general and can be extended to other material systems, including PEEK/CF, with appropriate material-specific calibrations and validations. Nevertheless, the proposed sequential DEM-FEM methodology for process window determination is inherently general and transferable to a wide range of materials, including polymer composites, provided that the necessary material-specific parameters and validation data are available.

#### 2.2.1. DEM for Powder Spreading

The DEM model used in this study is adopted from our previous work [24], where the model is comprehensively calibrated and experimentally validated. In that prior study, powder flowability tests (angle of repose measurements) were conducted for both PEEK and PEEK/CF powders, and the simulation results showed a maximum relative error of 4.89% compared with the experimental measurements. In addition, powder deposition experiments were performed, and the simulated powder bed morphologies were found to be in good agreement with optical microscopy observations. These validations confirmed the reliability of the DEM model in capturing the flow and spreading behavior of the tested powder systems. Therefore, the present study directly applies this validated model to investigate the effects of spreading parameters on powder layer quality, without repeating the calibration and validation procedures.

**Figure 5:**
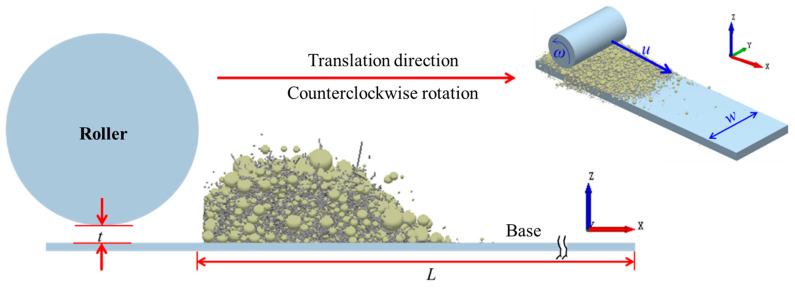
Model setup for the powder spreading simulation.

As shown in Figure 5, in the simulation model, the powder spreading system consists of three parts: the spreading roller (diameter D), the substrate (L × w), and the powder. Periodic boundary conditions are applied at the front and rear boundaries (Y direction, perpendicular to the *xz* plane) of the computational domain, thereby avoiding the influence of the substrate width w and the boundary effects of the geometry on the powder spreading outcome.

**Figure 6:**
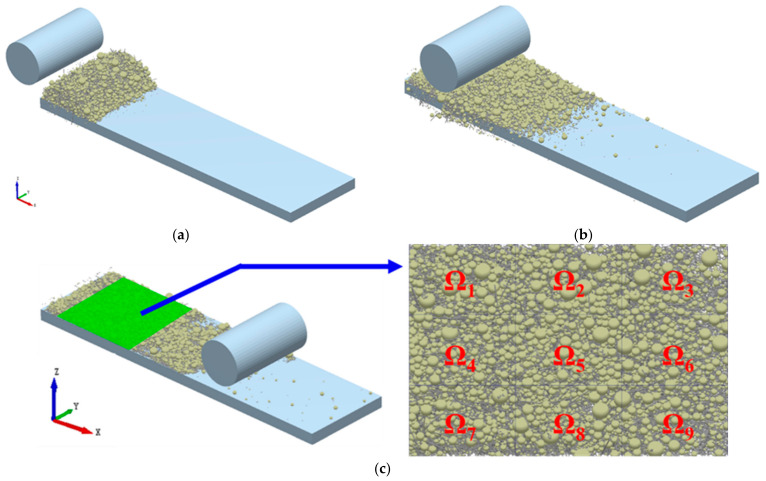
(**a**) Before powder spreading; (**b**) during powder spreading; (**c**) Powder layer state after spreading and the delineated zones for density analysis.

The roller spreading process is illustrated in Figure 6. First, a sufficient mass of particles is generated in front of the roller in the form of a static particle factory. Subsequently, the roller moves in the positive direction of the *x*-axis, accompanied by a counterclockwise rotation around its own central axis, finally completing the powder spreading process. It should be noted that the roller should rotate counterclockwise during spreading, which is consistent with actual practice. If clockwise rotation is adopted, powder particles would gradually enter the wedge-shaped space formed between the roller and the substrate plane, resulting in significant squeezing action between the roller and the particles, which would affect the already sintered powder layer. In contrast, during counterclockwise rotation, the particles move forward smoothly under the action of the roller, and the squeezing effect between the roller and the particles is relatively minor. To facilitate the study of the main factors influencing the powder spreading effect, the rotational speed of the spreading roller is set as a constant value. The distance t between the roller and the substrate represents the powder layer thickness. During spreading, the motion of the roller includes both translation and counterclockwise rotation (translational speed u, counterclockwise rotational speed ω). The main powder spreading parameters investigated in this paper are the layer thickness t and the translational speed u of the roller, where the translational speed of the roller is a primary factor affecting forming efficiency. The specific parameter design is shown in Table 4. Therein, the roller translational speed (u) is varied from 0.1 to 0.5 m/s with an increment of 0.1 m/s, covering the typical operational range for powder spreading processes while respecting the speed limit of our setup. The layer thickness range (0.10–0.15 mm) is selected based on the powder’s median particle size (D_50_ = 46.7 μm, Figure 3), corresponding to approximately 2.1–3.2 times D_50_, which is the recommended range for achieving uniform powder spreading. A step of 0.01 mm is used to capture the sensitive response of spreading quality to thickness variations within this range. The roller rotational speed (ω) is fixed at 5 rad/s, which is taken based on actual experience, as it is not a variable of interest in the current study.

The surface profile, density, and uniformity of density distribution of the powder layer are three important indicators for evaluating the quality of the spread powder layer. As shown in Figure 6c, upon completion of powder spreading, a specific region of the powder layer is selected to investigate its density information. This paper proposes a processing method that partitions and quantifies the powder layer. This approach allows for a more accurate comparison of the powder spreading effectiveness under different processes, improving the efficiency and accuracy of predicting the powder spreading process window. The target region is divided into nine sub-regions, Ω1–Ω9, in a 3 × 3 grid. The total particle mass within each sub-region is individually extracted, and the density of each region is calculated. The mean value and standard deviation of the densities of these nine regions can characterize the density magnitude and the uniformity of the density distribution of the current powder layer. The density evaluation region corresponds to the central stable area of the powder layer (excluding the edge regions influenced by boundary effects), which is divided into a 3 × 3 grid for the partitioned analysis.

#### 2.2.2. Thermo-Mechanical FEM Model

(1) Governing equations of the thermo-mechanical FEM model

In the three-dimensional homogeneous medium, the governing equation for heat conduction can be expressed as follows [46]:

(1) $$\rho C_{p}\cdot \partial T/\partial t=\nabla \cdot \left(k\nabla T\right)+Q$$

In the equation, T = T (t, x, y, z) represents the temperature at the corresponding temporal and spatial positions. ∂T/∂t denotes the rate of change in temperature with respect to time. ρ, k, and C_p_ represent the temperature-dependent density, thermal conductivity, and specific heat capacity, respectively, while Q denotes the energy input from the external heat source.

Based on the principle of heat conduction, once the temperature field is determined, information such as stress and strain can be obtained through the thermo-mechanical model. If a temperature difference ΔT (x, y, z) exists within the object, it will induce thermal expansion. The constitutive equation at this point will include an additional thermal expansion term and can be expressed as follows [47]:

(2) $$\left\{\begin{array}{l} \varepsilon_{xx}=\frac{1}{E}\left[\sigma_{xx}-\upsilon \left(\sigma_{yy}+\sigma_{zz}\right)\right]+\alpha_{T}\Delta T\\ \varepsilon_{yy}=\frac{1}{E}\left[\sigma_{yy}-\upsilon \left(\sigma_{xx}+\sigma_{zz}\right)\right]+\alpha_{T}\Delta T\\ \varepsilon_{zz}=\frac{1}{E}\left[\sigma_{zz}-\upsilon \left(\sigma_{yy}+\sigma_{xx}\right)\right]+\alpha_{T}\Delta T\\ \gamma_{xy}=\frac{1}{G}\tau_{xy},\gamma_{yz}=\frac{1}{G}\tau_{yz},\gamma_{zx}=\frac{1}{G}\tau_{zx} \end{array}\right.$$

where *α_T_* is the coefficient of thermal expansion; ε_xx_, ε_yy_, ε_zz_, γ_xy_, γ_yz_ and γ_zx_ represent the strains along different directions; and σ_xx_, σ_yy_, σ_zz_, τ_xy_, τ_yz_ and τ_zx_ denote the stresses along different directions. G is the shear modulus, E is the elastic modulus, and υ is Poisson’s ratio. Apart from the aforementioned constitutive equations, the remaining equilibrium equations, boundary conditions, and geometric equations are similar to those in ordinary elasticity problems.

(2) Heat source model

**Figure 7:**
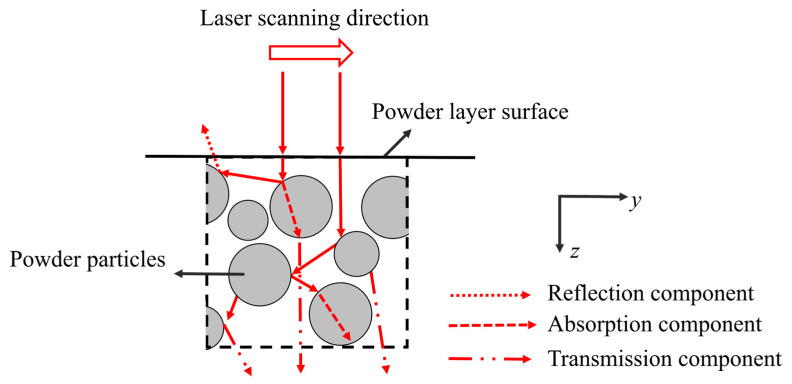
Schematic diagram of laser–powder interaction.

In PBF sintering simulations, the laser energy is often modeled as a surface heat source with a Gaussian distribution [48,49]. However, the interaction between the laser beam and the powder layer cannot be neglected [50,51]. When the laser beam irradiates the surface of the powder layer, the pores between the particles within the powder layer cause the laser beam to undergo multiple reflections and absorptions, as illustrated in Figure 7. The total laser energy is partitioned into three parts: the reflected part, the absorbed part, and the transmitted part. Only the absorbed portion of the laser energy is utilized for melting the powder. In addition to acting on the surface of the powder layer, the laser energy is also transferred to a certain depth within the powder layer. Therefore, besides considering the laser energy distribution on the powder layer surface, it is also necessary to account for the heat conduction along the depth direction within the powder layer. The Beer–Lambert attenuation law can be employed to describe the transmission of laser energy along the depth direction of the powder layer [16]. Consequently, the heat source equation can be expressed as follows:

(3) $$Q\left(x,y,z\right)=Q_{0}\left(x,y\right)\times \beta \times \exp\left(-\beta z\right)$$

In the equation, Q_0_ (W/m^2^) represents the laser energy distribution on the surface of the powder bed, β(m^−1^) denotes the attenuation coefficient, and the term exp(−βz) represents the attenuation form of the laser power along the depth direction [52]. Since the FEM model treats the powder layer as a homogeneous medium during modeling, it neglects the influence of the particle distribution within the powder layer on the laser absorptivity. Therefore, in the simulation calculations, β is set as a constant value, reflecting the average absorptivity of the laser energy by the powder layer. Subsequent experimental validation has confirmed that the results obtained from this approach are within an acceptable range. Q_0_ adopts the form of a Gaussian heat source distribution and can be expressed as follows:

(4) $$Q_{0}\left(x,y\right)=2P/\pi r_{laser}^{2}\cdot \exp\left(-2\left(x^{2}+y^{2}\right)/r_{laser}^{2}\right)$$

Therefore, the total heat input Q(W/m^3^) from the laser can be expressed as follows:

(5) $$Q\left(x,y,z\right)=2\beta P/\pi r_{laser}^{2}\cdot \exp\left(-2\left(x^{2}+y^{2}\right)/r_{laser}^{2}\right)\cdot \exp\left(-\beta z\right)$$

In the above equations, Q represents the total laser power input (W/m^3^), Q_0_ is the surface heat flux density (W/m^2^), β is the attenuation coefficient (m^−1^), P(W) is the laser power, and r_laser_(m) represents the spot radius. The attenuation coefficient β effectively incorporates the average absorptivity of the powder layer, as the powder layer is treated as a homogeneous medium in the FEM model.

Before sintering, the powder bed needs to be preheated to a specific temperature T_b_, which should be as close as possible to the melting point of the powder material. This approach not only reduces the required laser energy input but also helps avoid the generation of significant temperature differences during the sintering process. During sintering, the natural convection and thermal radiation between the powder layer surface and the surrounding environment can be expressed as follows:

(6) $$-k\frac{\partial T}{\partial \mu }=\delta \varepsilon \left(T^{4}-T_{\infty }^{4}\right)+c\left(T-T_{\infty }\right)$$

In the equation, k is the thermal conductivity, μ represents the unit normal vector, δ denotes the Stefan-Boltzmann constant (5.67 × 10^−8^ W·m^−2^·K^−4^), ε represents the emissivity (0.8 [53]), T_∞_ is the ambient temperature, and c is the natural heat convection coefficient.

It should be noted that while areal energy density is used as a convenient reference parameter for summarizing the process window, it represents only a first-order approximation. The same areal energy density can be achieved through different combinations of laser power and scanning speed, which may produce different peak temperatures and melt pool residence times. Therefore, the fitted relationships based on areal energy density are useful for practical guidance, but a more comprehensive thermal analysis considering the individual effects of laser power and scanning speed is recommended for future work.

All FEM simulations in this study are performed using Abaqus software 2014, with the moving laser heat source implemented via the DFLUX user subroutine.

#### 2.2.3. Method for Determining the Sintering Process Window

It should be noted that the coupling between the DEM and FEM models in this study is implemented in a sequential manner, where the DEM model is used to determine the optimal spreading parameters (especially the layer thickness) that yield a powder bed with optimized density, roughness, and uniformity. Only these optimized parameters are then transferred to the FEM model as input conditions. This simplification is justified by the fact that the DEM simulations serve as a prerequisite screening tool to ensure that the powder bed entering the sintering stage is sufficiently homogeneous, thereby supporting the uniform material property assumption adopted in the FEM thermal analysis. However, we acknowledge that under non-optimal spreading conditions, powder-bed heterogeneities such as localized porosity variations or surface irregularities could potentially affect the FEM predictions by altering local thermal conductivity and absorptivity. A more comprehensive strategy, e.g., mapping the spatially varying powder bed properties from DEM to FEM, would be valuable for capturing such effects and is recommended as a direction for future work. Based on the FEM model, this paper proposes a systematic methodology for determining the sintering process window. Based on the FEM model, this paper proposes a systematic methodology for determining the sintering process window.

The microscopic mechanism of sufficient sintering is analyzed, and the influence of various sintering process parameters (such as laser power, scanning speed, and hatching space) on the sintering process is characterized through the dimensions and temperature of the microscopic melt pool. This enables a more systematic and efficient determination of the sintering process window.

**Figure 8:**
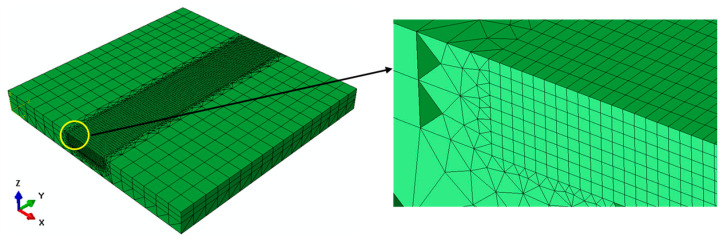
Mesh generation for the FEM model of the melt pool.

Figure 8 illustrates the mesh configuration of the FEM model used for melt pool calculation. The mesh in the sintering region is refined, whereas the mesh around the sintering trajectory is coarser. Specifically, the refined mesh size in the sintering region is 0.03 mm × 0.03 mm × 0.05 mm. For the simulation calculations, the analysis step is set to automatic mode, with each analysis step time designated as t′. The initial analysis step time scale is set to t′ × 10^−2^, and the minimum analysis step time scale is set to t′ × 10^−5^.

**Figure 9:**
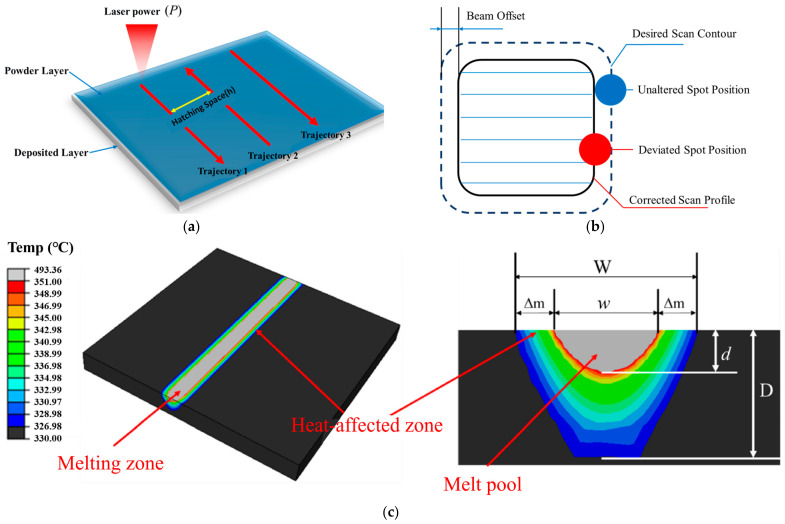
Schematic diagram of sintering process parameters and melt pool dimensions: (**a**) Laser power, scanning speed, and hatching space; (**b**) Beam offset; (**c**) Melt pool dimensions and HAZ.

The contour parameters and internal parameters of the sintering region influence the surface properties and mechanical properties of the formed parts, respectively. As shown in Figure 9a,b, the contour parameters include beam offset, scanning speed, and laser power; the internal parameters include laser power, scanning speed, and hatching space.

The sintering process window is determined through the dimensions and temperature information of the melt pool, as illustrated in Figure 9c. During the calculation, the sintering temperature field is first obtained, and the region where the temperature exceeds the melting point (the endpoint of the melting region) defines the extent of the melt pool. In Figure 9c, w and d represent the effective melt pool width and depth, respectively. To ensure sufficient melting of the powder layer, constraint criteria for sufficient sintering of the powder layer need to be established, with the specific analysis as follows. It is worth noting that the threshold values used in the following criteria (i.e., 1.5t and 2t) are determined based on the physical interpretation of melt pool depth relative to powder layer thickness. These values are established through extensive parametric simulations and represent a generalizable trend rather than case-specific observations. The 1.5t threshold provides a 50% penetration margin beyond the layer thickness, serving as a practical safety buffer to accommodate process fluctuations and ensure consistent interlayer bonding. The 2t upper boundary corresponds to the onset of significant over-sintering, beyond which thermal accumulation and melt pool instability become pronounced. These thresholds are consistently observed across a wide range of laser powers and scanning speeds, and their validity is further confirmed by the subsequent experimental verification.

(1) If d < t (where t is the powder layer thickness), the laser energy fails to penetrate the powder layer, resulting in insufficient melting of the powder layer. This may even lead to interlayer delamination and sintering failure.

(2) If t < d < 1.5t, sufficient melting of the powder layer can generally be ensured. However, due to the instability inherent in the sintering process, occasional insufficient sintering may occur, affecting the mechanical properties of the formed parts.

(3) If 1.5t < d < 2t, this represents an ideal condition. It avoids incomplete sintering while preventing the occurrence of over-sintering defects. Therefore, for the interior region of the sintered contour, a melt pool depth of 1.5t is considered ideal.

(4) If d > 2t, over-sintering defects are likely to occur, which will adversely affect the mechanical properties of the formed parts.

Within the extent of the heat-affected zone (HAZ, denoted as Δm in Figure 9c), the powder material is in a “semi-molten” state, which can significantly affect the accuracy of the formed parts. As shown in Figure 9c, a cross-section of the temperature field along the scanning trajectory is intercepted. In this figure, the black region represents the preheating temperature field of the powder bed, while the grey region denotes the area where the temperature exceeds the melting point of the material. The material in the region between the black and grey zones, where the temperature lies between the preheating temperature and the melting point, is in a semi-molten state. The unilateral width, denoted as Δm, of this intermediate region is used to characterize the size of the HAZ. To minimize the impact of this “semi-molten” state, the width of the HAZ (Δm) should be as small as possible. Figure 9b is a schematic diagram of the beam offset. The boundary of the laser spot (red area) should be tangent to the actual contour to reduce dimensional deviations caused by over-sintering; therefore, the beam offset should equal half the melt pool width. In the interior region of the sintering area, the hatching space h between scanning trajectories should be smaller than the melt pool width w to avoid areas of insufficient melting within the powder layer. Furthermore, the maximum melt pool temperature T_max_ should lie within the stable sintering region (SSR, which is the temperature range ensuring sufficient sintering of the powder layer, spanning from the endpoint of the material melting temperature range Tmf to the onset point of material decomposition temperature T_ds_) [54]. Finally, the aforementioned constraint criteria for sufficient sintering are summarized in Table 5.

The relationship between melt pool dimensions/temperature and laser energy density is key to determining process parameters. In this study, the areal energy density is employed for calculation, which can be expressed as

(7) $$E_{P}=\frac{P}{\phi_{laser}v}$$

where P is the laser power, v is the scanning speed, and Φ_laser_ is the laser spot diameter (taken as 0.6 mm).

## 3. Results and Discussion

### 3.1. Numerical Investigation of Powder Spreading

This section investigates the microscopic mechanism of the powder spreading process for PEEK and PEEK/CF powders. A research methodology is proposed to evaluate powder layer performance based on surface profile, density magnitude, and density distribution uniformity. The effects of spreading speed and layer thickness are analyzed, and the influence of the reinforcement phase on the powder spreading mechanism and powder layer performance is examined. The optimal powder layer thickness for the PEEK material is determined.

#### 3.1.1. Effect of Powder Spreading Speed

**Figure 10:**
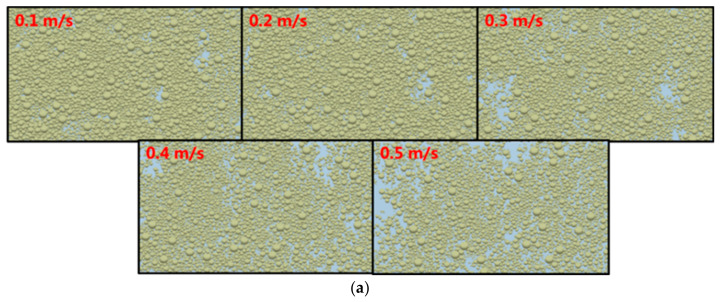

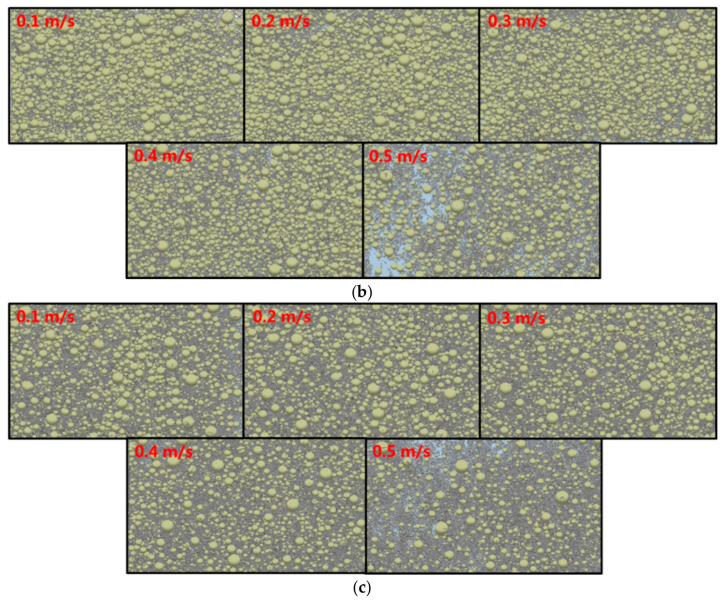
Apparent characteristics of powder layers for PEEK and PEEK/CF powders under different spreading speeds: (**a**) PEEK; (**b**) PEEK/CF_30wt %; (**c**) PEEK/CF_50wt %.

The spreading speed (translational velocity) of the roller significantly influences the forming efficiency of PBF. When investigating the effect of spreading speed, the powder layer thickness is initially controlled at 0.12 mm. The apparent characteristics of the powder layers for PEEK and PEEK/CF powders at different roller translational speeds (0.1 m/s to 0.5 m/s) are shown in Figure 10. For the PEEK powder layer, the apparent porosity increases noticeably with increasing spreading speed. In contrast, for the PEEK/CF powder layer, except for the more pronounced apparent porosity at the speed of 0.5 m/s, the differences in apparent porosity under the other spreading speeds are relatively small.

**Figure 11:**
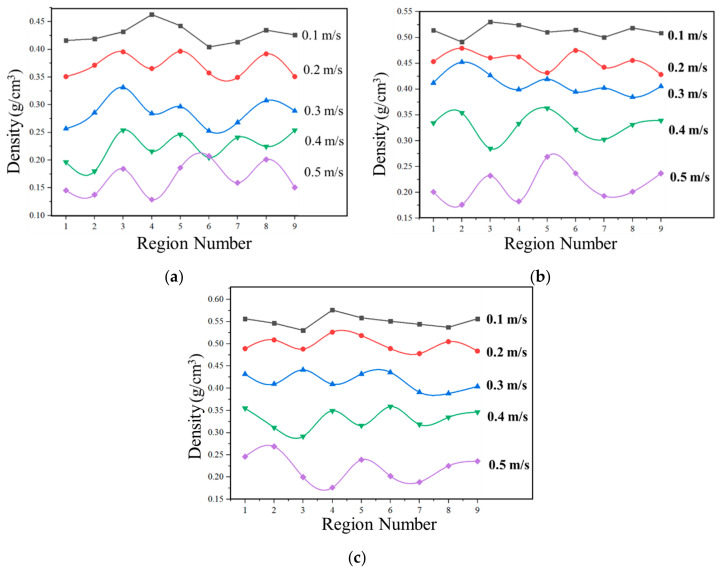
Density of each region in the powder layer, divided into a 3 × 3 grid (nine zones), under different spreading speeds: (**a**) PEEK; (**b**) PEEK/CF_30wt %; (**c**) PEEK/CF_50wt % (the numbers at the end of each curve indicate the spreading speed (m/s)).

**Figure 12:**
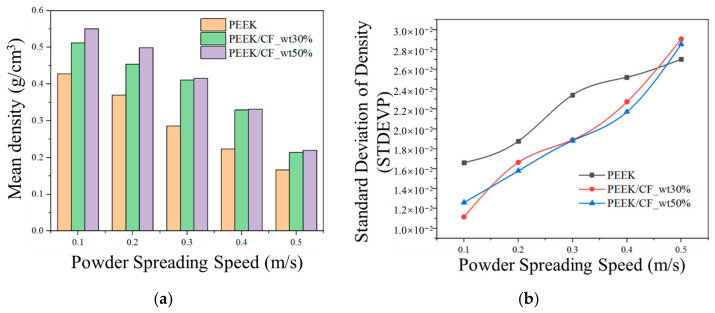
Effect of spreading speed on the average density (density magnitude) and standard deviation (density uniformity) across nine zones of the powder layer: (**a**) Effect of spreading speed on the average density of the powder layer; (**b**) Effect of spreading speed on the standard deviation of density in the powder layer.

To quantitatively characterize the density information of the powder layer, the powder layer is divided into nine regions in a 3 × 3 grid, and the density of each region is calculated, as shown in Figure 11. It can be observed that for both PEEK and PEEK/CF materials, as the spreading speed increases, the density of the powder layer decreases and its distribution becomes more non-uniform. The mean value and standard deviation of the densities of the nine regions characterize the average density and the uniformity of the density distribution of the current powder layer, respectively, as illustrated in Figure 12. For PEEK and PEEK/CF materials, the average density of the powder layer is negatively correlated with the spreading speed, while the standard deviation of the powder layer density distribution is positively correlated with the spreading speed. At the same spreading speed, the average density of the PEEK/CF powder layer is greater than that of the PEEK material. As the CF mass fraction increases, the average density of the powder layer increases, because the solid density of CF is higher than that of the PEEK material. Meanwhile, the difference in average density between the PEEK and PEEK/CF powder layers gradually decreases with increasing spreading speed, although this difference remains significant. In contrast, the difference in average density between the composite powders PEEK/CF_30wt % and PEEK/CF_50wt % decreases markedly with increasing spreading speed, and when the spreading speed is equal to or greater than 0.3 m/s, their average densities are similar. Furthermore, the magnitude and trend of the standard deviation of density for the two PEEK/CF composite powder layers are similar. Except at a spreading speed of 0.5 m/s, the standard deviation of density for the PEEK/CF powder layers is significantly smaller than that for the PEEK material under the other spreading speeds.

Therefore, for PEEK and PEEK/CF materials, a lower spreading speed is conducive to achieving favorable powder layer performance (characterized by high average density and uniform distribution). As the spreading speed gradually increases, although the powder layer performance deteriorates, the extent of this deterioration is significantly smaller for the PEEK/CF powder layer compared to the PEEK material. This indicates that the addition of CF particles to the PEEK powder mitigates the trend of deteriorating powder layer performance (in terms of density magnitude and distribution) caused by higher spreading speeds. When the mass fraction of CF in the PEEK/CF powder increases, this mitigating effect does not exhibit significant differences with further increases in spreading speed. It should be noted that the higher average density of PEEK/CF layers compared with PEEK layers is partly attributable to the higher solid density of CF particles. Therefore, direct comparison of mass density values between PEEK and PEEK/CF should be interpreted with caution. However, the density trends within each material system, which show a decrease in density and the deterioration of uniformity with increasing spreading speed, remain valid and reflect the effect of process parameters on packing efficiency. Since the sintering process window established in this study is based on PEEK material only, the potential density ambiguity between PEEK and PEEK/CF does not affect our main conclusions.

**Figure 13:**
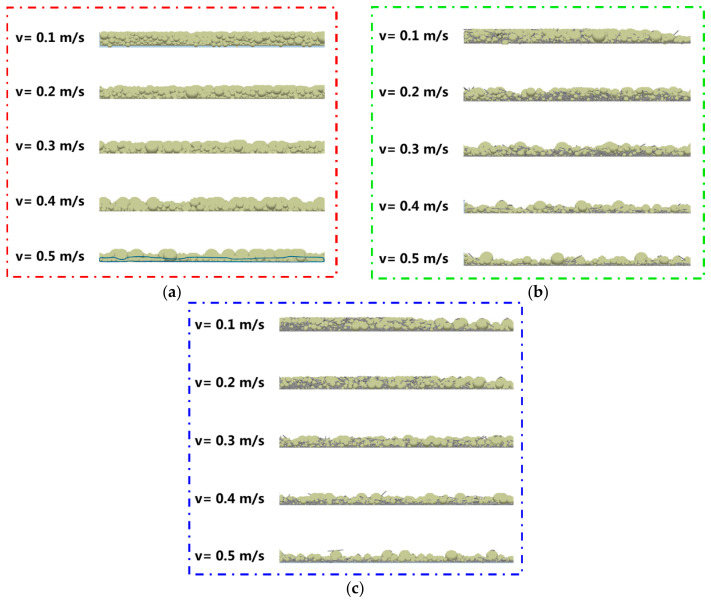
Effect of spreading speed on the surface profile of the powder layer: (**a**) PEEK; (**b**) PEEK/CF_30wt %; (**c**) PEEK/CF_50wt %.

Surface profile is another crucial indicator for evaluating powder layer performance. The qualitative influence of spreading speed on surface profile can be intuitively assessed based on the contour of particles on the powder layer surface. Figure 13 presents cross-sectional views of the powder layer obtained from DEM simulations. These views are used for qualitative observation of particle distribution and surface contour under different spreading speeds, providing mechanistic insights into the deposition behavior of particles. Quantitative evaluations of powder layer quality, including density and uniformity, are provided separately in the subsequent parametric analysis. As shown in Figure 13, when the powder layer thickness is constant, the surface profile of both PEEK and PEEK/CF powder layers increases with increasing spreading speed. For the PEEK powder layer, at higher spreading speeds, larger particles are distributed more dispersedly on the powder layer surface, while smaller particles are distributed more uniformly in the lower part of the powder layer (blue circled areas in Figure 13a). These larger, dispersed particles are the primary cause of increased surface profile. For the PEEK/CF composite powder, higher spreading speeds lead to a more dispersed distribution of larger particles (PEEK and larger CF particles) on the powder layer surface, resulting in greater roughness. This phenomenon can be further analyzed. As spreading speed increases, small particles are deposited relatively uniformly at the bottom of the powder layer, whereas large particles become more dispersedly distributed on the surface, significantly impacting surface profile. This occurs because, during roller spreading, small particles have a shorter contact time with the roller surface and deposit more quickly into the gap between the roller and the substrate. In contrast, larger particles experience longer contact with the roller surface, making them more prone to being carried forward by the roller motion, which hinders their deposition. Besides PEEK particles, the large particles distributed on the powder layer surface also include CF particles with a high aspect ratio. Similarly, due to the difficulty these large CF particles face in rapidly depositing into the wedge-shaped gap between the roller and substrate during spreading, they tend to be carried forward by the roller, ultimately affecting the surface profile of the powder layer. Therefore, for PEEK/CF composite powders, a higher spreading speed is more likely to negatively impact the surface profile of the powder layer.

#### 3.1.2. Effect of Powder Layer Thickness

The investigation into the influence of spreading speed on powder layer performance reveals that a lower spreading speed is beneficial for achieving ideal powder layer characteristics. However, an excessively low spreading speed can reduce forming efficiency. Therefore, it is necessary to consider other spreading parameters, such as powder layer thickness, to balance powder layer performance against forming efficiency and strive to select the optimal parameter combination.

**Figure 14:**
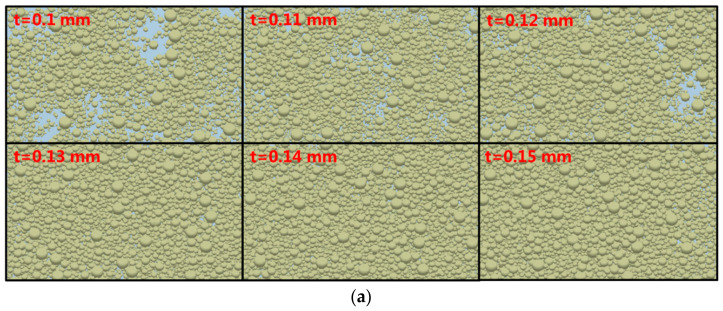

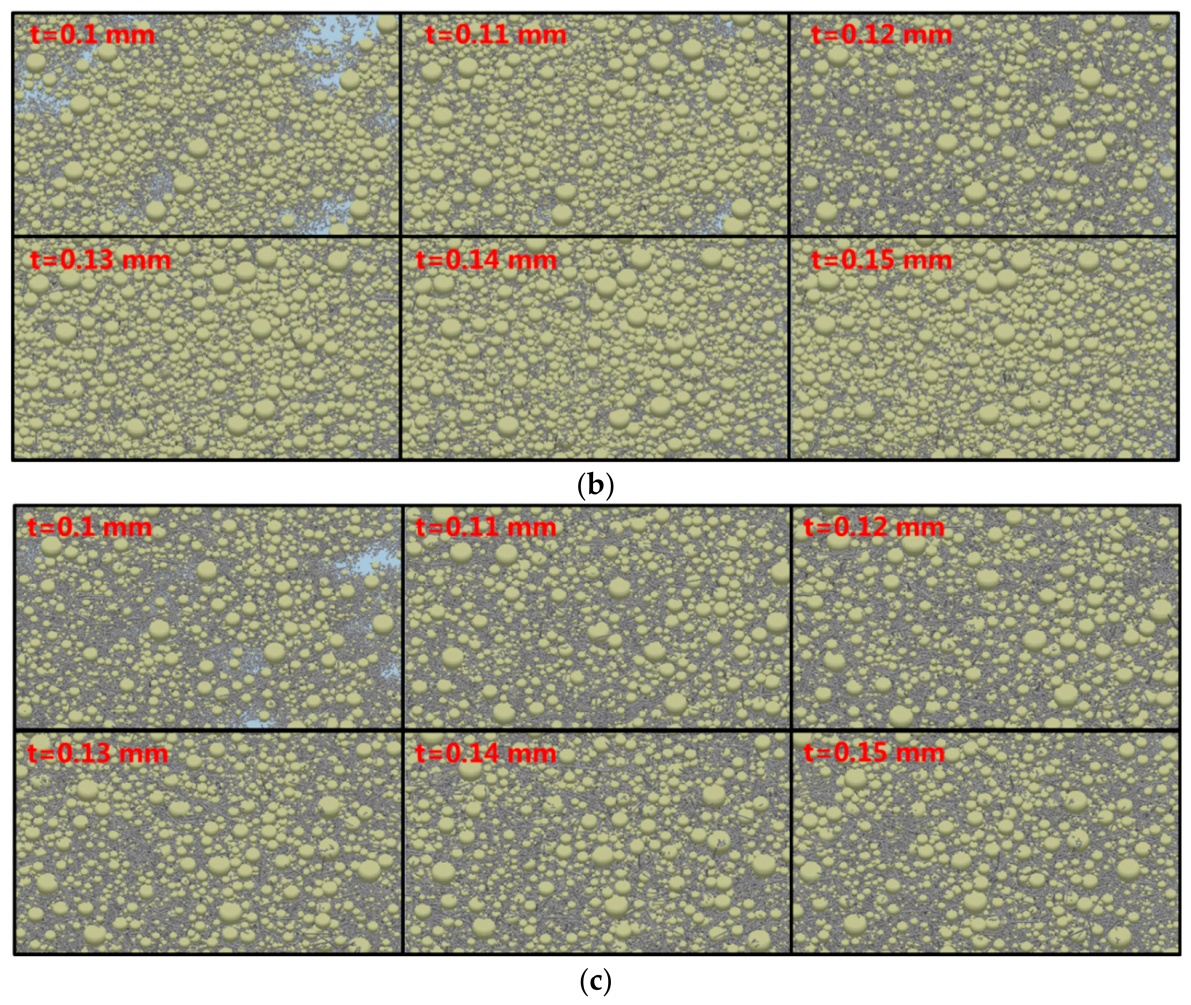
Apparent characteristics of powder layers for PEEK and PEEK/CF powders under different layer thicknesses: (**a**) PEEK; (**b**) PEEK/CF_30wt %; (**c**) PEEK/CF_50wt %.

When investigating the powder layer thickness, the spreading speed is set to 0.2 m/s. The apparent characteristics of the powder layers for PEEK and PEEK/CF composite powders at different layer thicknesses (0.1 mm to 0.15 mm) are shown in Figure 14. For the PEEK powder layer, when the layer thickness is less than 0.13 mm, there are noticeable apparent pores. In contrast, for the two types of PEEK/CF powder layers, no obvious apparent pores are present when the layer thickness is greater than 0.1 mm.

**Figure 15:**
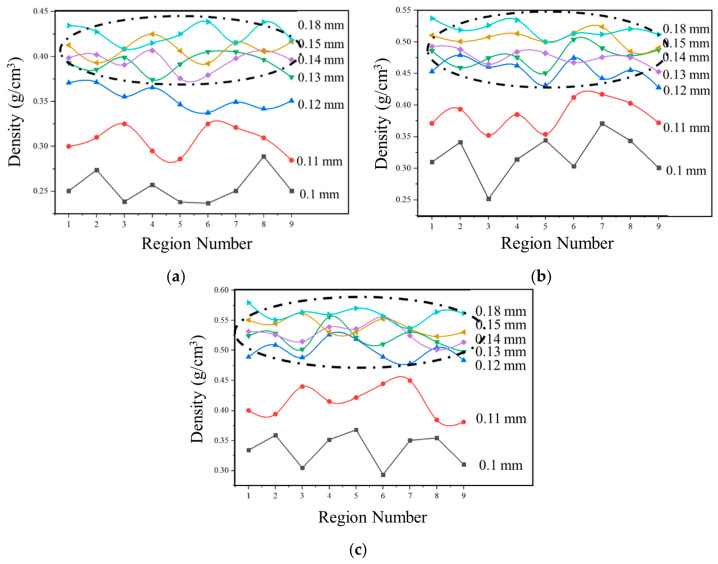
Density of each region in the powder layer, divided into a 3 × 3 grid (nine zones), under different layer thicknesses: (**a**) PEEK; (**b**) PEEK/CF_30wt %; (**c**) PEEK/CF_50wt % (the numbers at the end of each curve indicate the layer thickness (mm)).

**Figure 16:**
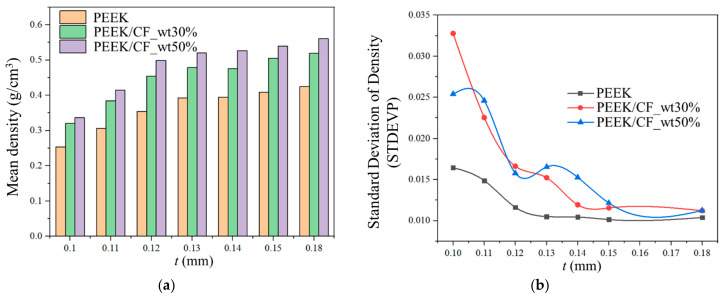
Effect of layer thickness on the average density (density magnitude) and standard deviation (density uniformity) across nine zones of the powder layer: (**a**) Effect of layer thickness on the average density of the powder layer; (**b**) Effect of layer thickness on the standard deviation of density in the powder layer.

To quantitatively characterize the density information of the powder layer, the powder layer is divided into nine regions in a 3 × 3 grid, and the density of each region is calculated, as shown in Figure 15. For PEEK and both types of PEEK/CF materials, as the powder layer thickness increases, the density of the powder layer also increases. Specifically, when the layer thickness is equal to or greater than 0.13 mm, the density of the PEEK powder layer and the uniformity of its density distribution exhibit similar characteristics; when the layer thickness exceeds 0.12 mm, the density of the PEEK/CF powder layers and the uniformity of their density distribution show similar behavior (indicated by the dashed circle in Figure 15). The mean value and standard deviation of the densities of the nine regions of the powder layer characterize the average density and the uniformity of the density distribution of the current powder layer, respectively, as illustrated in Figure 16. For PEEK and PEEK/CF materials, as the powder layer thickness increases, the average density of the powder layer increases, although the increasing trend tends to plateau. When the layer thickness is less than or equal to 0.13 mm, the standard deviation of the powder layer density distribution decreases with increasing layer thickness, indicating that the uniformity of the powder layer density distribution is positively correlated with layer thickness. When the layer thickness exceeds 0.13 mm, the differences in the uniformity of the powder layer density distribution are relatively small, and a favorable state can generally be achieved. Therefore, an excessively small layer thickness is more likely to cause a deterioration in powder layer performance (density magnitude and distribution), while also leading to an increased number of spreading passes and reduced forming efficiency. It is worth noting that as the powder layer thickness increases, both the density magnitude and uniformity improve, and the powder layer density approaches the tap density of the powder. Furthermore, although the density magnitude and uniformity of the powder layer improve with increasing layer thickness, an excessively large layer thickness results in higher consumption of raw material powder, which is detrimental to cost control. During the subsequent sintering process, if the layer thickness is too large, the powder layer may not be sufficiently sintered, leading to interlayer sintering defects or even forming failure. Additionally, the staircase effect caused by a larger layer thickness significantly impacts the accuracy of the formed parts. Therefore, when evaluating the appropriate layer thickness based on powder layer performance (density magnitude and distribution), the minimum layer thickness that satisfies the powder spreading conditions should be selected.

**Figure 17:**
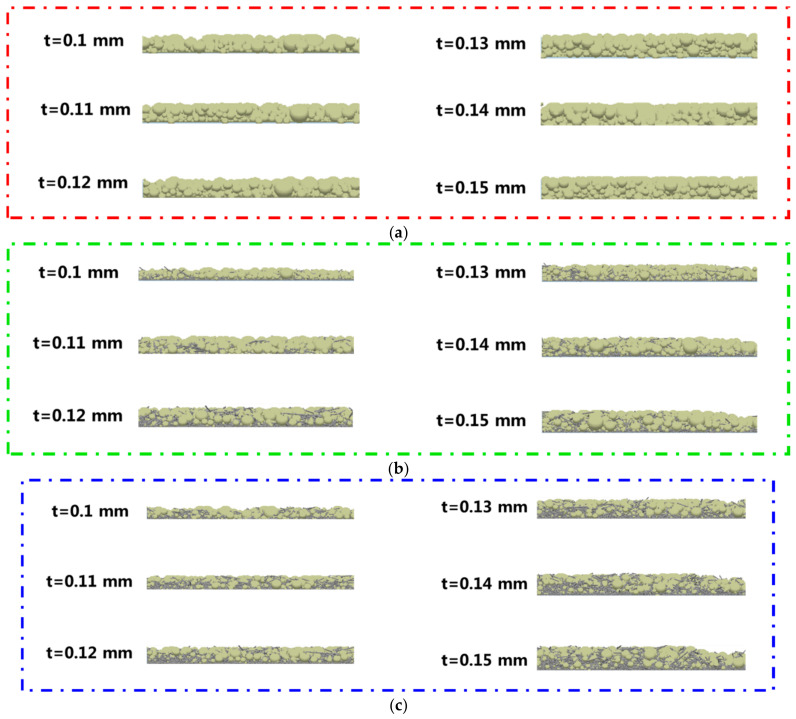
Effect of layer thickness on the surface profile of the powder layer: (**a**) PEEK; (**b**) PEEK/CF_30wt %; (**c**) PEEK/CF_50wt %.

Based on the contour of particles on the powder layer surface, the qualitative influence of powder layer thickness on surface profile can be intuitively assessed, as shown in Figure 17. For the PEEK material, when the layer thickness is equal to or greater than 0.13 mm, the surface profile of the powder layer is considered ideal. For the PEEK/CF material, the powder layer surface is relatively rough only when the layer thickness is 0.1 mm; for the remaining layer thicknesses, the surface profile of the powder layer is relatively ideal. This indicates that a smaller layer thickness is more likely to adversely affect the surface flatness of the powder layer.

**Figure 18:**
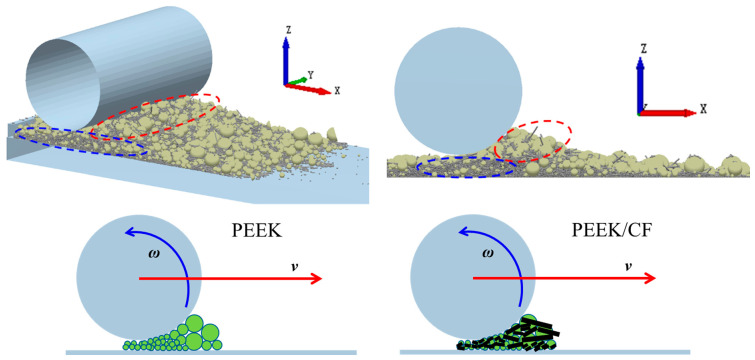
Schematic diagram showing the contact and relative positions of large and small particles with the roller during powder spreading.

As illustrated in Figure 18, when the layer thickness is relatively small, fine particles have a shorter contact time with the roller during spreading and quickly enter the gap between the roller and the substrate. In contrast, larger particles (PEEK particles and larger CF particles) experience a longer contact time with the roller, making them more prone to being carried forward by the roller motion. This leads to difficulties in particle deposition and results in a non-uniform distribution. These unevenly distributed large particles are the direct cause of increased surface profile in the powder layer. As the layer thickness gradually increases, the proportion of large particles that have a long contact time with the roller and experience deposition difficulties diminishes. The vast majority of particles are able to deposit rapidly into the gap between the roller and the substrate, consequently resulting in a smoother powder layer surface.

#### 3.1.3. Analysis of Powder Spreading Mechanisms

**Figure 19:**
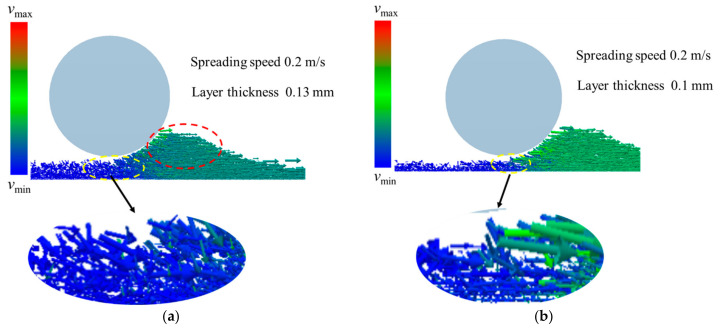
Velocity distribution of PEEK powder particles during the spreading process: (**a**) Spreading speed: 0.2 m/s, layer thickness: 0.13 mm; (**b**) Spreading speed: 0.2 m/s, layer thickness: 0.1 mm.

In this section, the influence of the roller on powder particles during the spreading process is comprehensively analyzed through the velocity distribution of the particles. The velocity distribution field of PEEK powder particles at a spreading speed of 0.2 m/s and a layer thickness of 0.13 mm is shown in Figure 19a (the length and direction of the arrows in the figure represent the magnitude and direction of the velocity). Particles in the upper part of the powder pile (area circled in red), upon contact with the roller, have a velocity direction consistent with the roller’s spreading direction. The higher the spreading speed, the greater the forward velocity of particles in this region, making downward deposition more difficult. Therefore, the translational speed of the roller should not be excessively high. In the area directly beneath the roller (circled in yellow), the velocity of the vast majority of particles reaches a minimum (stationary state), indicating that the powder in this region has essentially completed the deposition process. Figure 19b shows the velocity distribution field of powder particles at a spreading speed of 0.2 m/s and a layer thickness of 0.1 mm. The velocity of particles in the upper part of the powder pile is similar to the case with a layer thickness of 0.13 mm, with particle velocities being primarily aligned with the spreading direction. However, compared to the 0.13 mm layer thickness, the proportion of powder particles reaching the minimum velocity in the area directly beneath the roller (yellow circle) decreases (the proportion of blue arrows decreases), indicating that a small fraction of particles in this region still possess forward motion. This inevitably affects the particle deposition effectiveness and subsequently impacts the powder layer performance.

**Figure 20:**
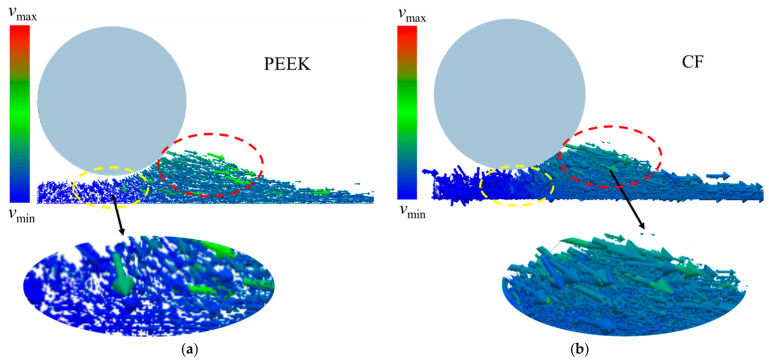
Velocity distribution of PEEK and CF particles in PEEK/CF powder at a spreading speed of 0.2 m/s and a layer thickness of 0.13 mm: (**a**) PEEK particles; (**b**) CF particles.

At a spreading speed of 0.2 m/s and a layer thickness of 0.13 mm, the velocity distribution field of PEEK/CF composite powder particles is calculated. For ease of analysis, the velocity fields of PEEK particles and CF particles within the PEEK/CF composite powder are discussed separately, as shown in Figure 20. It can be observed that the velocity of PEEK particles in the upper part of the powder pile (area circled in red) is directed obliquely downward, indicating that while moving along the spreading direction, these particles also exhibit a tendency for downward deposition. This differs from the velocity field of powder particles in the pure PEEK material (Figure 19). In the area beneath the roller (yellow circle), most PEEK particles have reached a stationary state, indicating completed spreading. Figure 20b shows the velocity field distribution of CF particles within the PEEK/CF powder during spreading. The density of velocity vectors for CF particles is significantly greater than that for PEEK particles. The velocity direction of CF particles in the upper part of the powder pile (red circled area) is also obliquely downward, suggesting that these particles possess a tendency for downward deposition while moving along the spreading direction. This downward deposition tendency is likely attributable to the morphology of the CF particles. For spherical particles, the contact point with the roller remains stable at any position during spreading, and they tend to move along the spreading direction under the influence of the roller’s translation and rotation. However, for cylindrical CF particles with varying lengths, the contact position with the roller during spreading is random, making it less likely for them to move uniformly along the spreading direction under the roller’s influence. Consequently, during the spreading process of the PEEK/CF composite powder, the vast majority of CF particles tend to move obliquely downward. Furthermore, due to the large quantity and small size of CF particles, their movement tendency can readily influence the PEEK particles. It is worth noting that the present study focuses on macroscopic powder layer quality indicators to evaluate the effect of CF addition on spreading behavior, without performing detailed particle-scale tracking of CF orientation, segregation, or anisotropy. These factors could provide additional mechanistic insights and may vary with fiber size, loading, or roller kinematics. However, the consistent trend observed across two different CF loadings (30 wt % and 50 wt %) and multiple spreading speeds suggests that the effect of CF addition in slowing the deterioration of layer quality at higher speeds is robust within the investigated parameter range. Detailed CF-scale analyses are recommended as future work.

#### 3.1.4. Determination of the Powder Spreading Process Window

**Figure 21:**
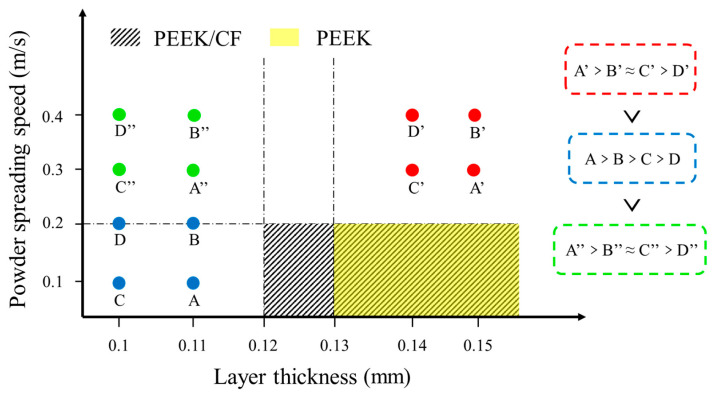
Process window for powder spreading speed and layer thickness.

To intuitively illustrate the differences in spreading parameters and their effects for PEEK and PEEK/CF powders, Figure 21 summarizes the powder spreading process window targeting roller translational speed and powder layer thickness. The reasonable parameter regions for PEEK and PEEK/CF powders are represented by yellow and hatched backgrounds, respectively. The reasonable spreading speed for both is 0.1–0.2 m/s, while the reasonable layer thickness range for PEEK/CF powder is slightly broader than that for PEEK powder. This indicates that the addition of CF particles to the PEEK powder can lower the minimum feasible powder layer thickness, thereby mitigating the staircase effect in formed parts and improving their dimensional accuracy. The upper-left, upper-right, and lower-left regions of the parameter window correspond to areas where either spreading speed or layer thickness is unreasonable. For the lower-left region of the parameter window, four sets of parameter points (A, B, C, D) are selected for powder spreading process simulation. Powder layer performance is still evaluated based on surface profile, density, and density distribution uniformity. According to the calculation results, the comprehensive performance of the powder layers corresponding to the four parameter points, ranked in descending order, is A > B > C > D. This indicates that layer thickness has a greater impact on powder layer performance, followed by spreading speed. Even with a relatively high spreading speed, the powder layer performance at a larger layer thickness can still be superior to that achieved with a small layer thickness and a low spreading speed. For the upper-right region of the parameter window, the comprehensive performance of the powder layers corresponding to the four parameter points, ranked in descending order, is A’ > B’ ≈ C’ > D’. This region is characterized by relatively large layer thicknesses, and the differences in powder layer performance among the four parameter points are less pronounced than those in the lower-left region. Notably, the powder layer performances corresponding to parameters B’ and C’ are similar, suggesting that when the layer thickness is within a reasonable range, a higher speed does not lead to significant differences in powder layer performance. Therefore, when the layer thickness meets certain requirements, the spreading speed can be appropriately increased to enhance forming efficiency. For the upper-left region of the parameter window, where both spreading speed and layer thickness parameters are unsuitable, the corresponding powder layer performance is uniformly poor. Excluding the lower-right reasonable parameter region, the optimal powder layer performance among the other regions is found in the upper-right region, followed by the lower-left region. This further confirms that during the powder spreading process, layer thickness exerts a greater influence on powder layer performance than spreading speed. However, an excessively large layer thickness can lead to the staircase effect, formed part defects, and increased raw material costs. Consequently, identifying the optimal powder layer thickness through powder spreading process simulation is of paramount importance.

Through the investigation of the powder spreading process, the optimal powder layer thickness for PEEK is determined to be 0.13 mm, while that for PEEK/CF powder is found to be 0.12 mm. This value is slightly smaller than that for PEEK powder, which positively impacts the reduction in the staircase effect in formed parts. Under these optimal layer thicknesses, the powder layer performance is considered ideal, effectively minimizing the negative influence of the powder layer on the subsequent sintering process. This also serves as a prerequisite for subsequently determining the sintering process window.

### 3.2. Sintering Process Window

**Figure 22:**
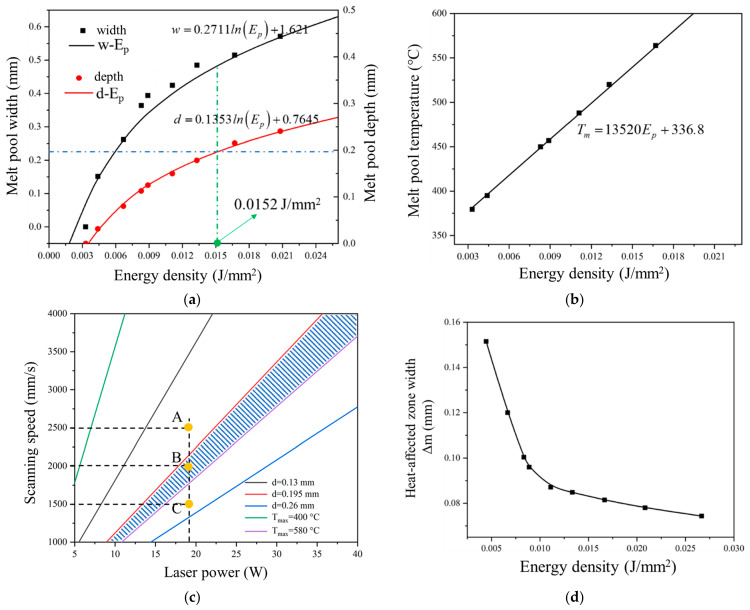
Sintering process window for PEEK: (**a**) Relationship between melt pool width/depth and energy density; (**b**) Relationship between melt pool temperature and energy density; (**c**) Parameter window for laser power and scanning speed; (**d**) Relationship between HAZ width and energy density.

It should be noted that the PEEK/CF material is employed only in the powder spreading simulations to investigate the particle-scale behavior of reinforced powders, while all experimental tensile tests in this study are conducted on pure PEEK specimens to optimize the sintering process parameters. Using the optimal layer thickness (0.13 mm) determined from the PEEK powder spreading process study as an input condition, the relationship between melt pool dimensions/temperature and energy density is calculated to establish a reasonable sintering process window, thereby addressing the PBF process challenges for PEEK material. Employing the combinations of laser power and scanning speed parameters shown in Table 6 (initial parameter design for the calculations is performed by arranging parameter combinations in order of increasing energy density, based on literature or empirical printing experience), the functional relationships between energy density E_p_ and the melt pool width w, depth d, maximum temperature T_max_, and HAZ width Δm are calculated, respectively. These relationships are presented in Figure 22.

Figure 22a illustrates the relationship between the melt pool width w and depth d with the energy density Ep, which can be fitted using the following expressions.

(8) $$w=0.2711\ln\left(E_{p}\right)+1.621\;(R\text{-}\mathrm{square}:\;0.9317)$$

(9) $$d=0.1353\ln\left(E_{p}\right)+0.7645\;(R\text{-}\mathrm{square}:\;0.9964)$$

Figure 22b depicts the relationship between the maximum melt pool temperature T_max_ and the energy density E_p_, which can be fitted using a linear relationship as follows:

(10) $$T_{m}=13520E_{p}+336.8\;(R\text{-}\mathrm{square}:\;0.9996)$$

Based on Equations (8)–(10) and incorporating the sufficient sintering constraints from Table 5, linear relationship diagrams between laser power and scanning speed are plotted, as shown in Figure 22c. These are generated by controlling the melt pool depth to 0.13 mm, 0.195 mm, and 0.26 mm, and the melt pool temperature to 400 °C and 500 °C (both within the SSR range). The shaded region in the figure represents the reasonable parameter interval for laser power and scanning speed. Figure 22d shows the relationship between the HAZ width and energy density, which are negatively correlated. Therefore, while satisfying other sintering constraints, to minimize the HAZ width, the energy density in the contour region should be as high as possible. Consequently, contour parameters should be selected along the purple line in Figure 22c. At this point, the corresponding melt pool width is 0.532 mm; thus, the beam offset γ can be determined as 0.266 mm. For the interior region, while meeting other sintering constraints, the energy density corresponding to a melt pool depth of 1.5t is generally considered optimal, represented by the red line in Figure 22c. The corresponding laser energy density is 0.015 J/mm^2^, and the associated melt pool width is 0.48 mm; therefore, the hatching space should be less than 0.48 mm. It can be observed that the sintering process window for the PEEK material is notably narrow. When selecting specific laser power and scanning speed values, the actual equipment capabilities should be considered. Provided the energy density requirements are met, a higher scanning speed leads to greater process efficiency.

Therefore, by combining the sufficient sintering constraint conditions and the fitted equations, the sintering process window for the scanned contour and interior region of the PEEK material can be determined and is summarized in Table 7.

### 3.3. Experimental Validation

The sintering process window for PEEK is experimentally validated through the mechanical properties and relative density of the material. The laser power employed is 19 W, with scanning speeds of 2500 mm/s, 2000 mm/s, and 1500 mm/s, corresponding to parameter points A, B, and C in Figure 22c, respectively. Among these, parameter point B lies within the laser parameter window, while parameter points A and C lie outside the window. The hatching space is set to 0.2 mm.

The density test specimens are cubes with dimensions of 10 mm × 10 mm × 4 mm. Three specimens are tested for each parameter set, and each specimen is measured three times. The average value is taken as the target density for that specimen. The relative density of a specimen is the ratio of the measured density to the solid density, expressed as:

(11) $$\rho_{\mathrm{rel}}=\frac{\rho_{\mathrm{mea}}}{\rho_{\mathrm{PEEK}}}\times 100\%$$

where ρ_rel_ is the relative density of the specimen, ρ_mea_ is the measured density of the specimen, and ρ_PEEK_ is the solid density of the PEEK material (taken as 1.3 g/cm^3^).

The mechanical properties are tested in accordance with the national standard GB/T 1040.2-2022 [55], utilizing the 1BA type specimen. Five specimens are tested for each parameter set, and the average value is taken as the target tensile strength [56].

The measured density and relative density results under different process parameters are presented in Table 8.

For parameter point B, where the energy density lies within the sintering process window, its maximum relative density reached 99.31%, which is superior to that of the other parameter groups.

The tensile strengths of the specimens for different parameter groups are shown in Table 9. It can be observed that when the scanning speed is 2000 mm/s (parameter point B, located within the sintering process window), the tensile strength of the specimens is greater than that of specimens corresponding to other scanning speeds. The tensile strength of specimens within the sintering process window is up to 13.1% higher than that of specimens outside the process window. Therefore, the PEEK sintering process window and the optimal parameter combination are reliable. For the three parameter sets investigated, the highest tensile strength was obtained at a scanning speed of 2000 mm/s (56.34 ± 2.59 MPa), which corresponds to the optimal condition predicted by the established process window. The lower scanning speed (1500 mm/s) and higher scanning speed (2500 mm/s) yielded lower tensile strengths (48.98 ± 6.18 MPa and 53.68 ± 5.29 MPa, respectively). The standard deviation for the window-internal condition (2.59 MPa) is smaller than those for the window-external conditions (6.18 MPa and 5.29 MPa), suggesting that the optimal condition also provides more consistent mechanical performance. We acknowledge that the experimental validation is limited to three parameter sets and does not include direct melt pool measurements. Nevertheless, the consistent agreement between the predicted optimal window and the observed tensile performance provides meaningful support for the practical utility of the established process window. Direct melt pool validation and quantitative porosity analysis are recommended for future work to further strengthen the validation.

**Figure 23:**
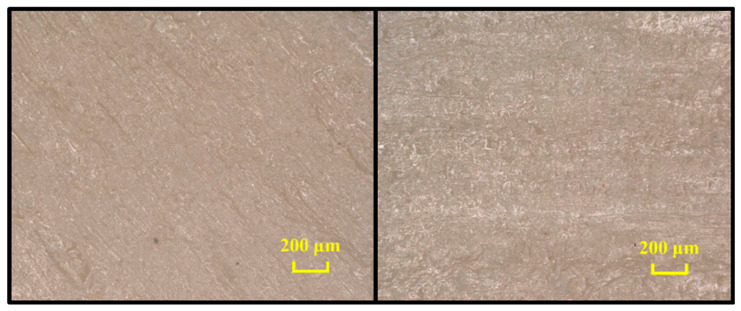
Microstructure of PEEK specimen processed within the optimal window. This image serves as a supplementary illustration; quantitative densification evidence is provided by the measured relative density (99.31%).

Secondly, the microstructure of the PEEK specimens is observed. When the tensile strength of PEEK reached its optimum (parameter point B), the cross-sectional microstructure of the PEEK specimen is examined using an ultra-depth three-dimensional microscope (magnification 150×; a clear image is available in the electronic version), as shown in Figure 23. It can be observed that the cross-sectional microstructure exhibits good uniformity, with no obvious defects such as pores being detected.

In summary, the reliability of the sintering process window is validated through relative density measurements, tensile tests, and microstructural observations of the PEEK material. The experimental validation is designed to test the effectiveness of the established process window by comparing three representative conditions: one inside the optimal window and two outside it. The results confirm that specimens processed within the window exhibit superior tensile strength, validating the predictive capability of the proposed simulation framework. Although the validation is limited to these three conditions, the simulation methodology itself has been extensively tested through parametric studies covering wide ranges of laser power, scanning speed, and layer thickness. The framework is general and can be extended to other powder materials and equipment with appropriate recalibration of material-specific parameters.

## 4. Conclusions

In this paper, a sequential DEM-FEM framework is constructed to conduct simulation studies on the powder spreading and sintering stages of PBF, forming a systematic simulation methodology. Compared with traditional trial-and-error approaches, this work predicts optimal powder spreading parameters more accurately and efficiently. Although PEEK is used as the representative material in this study, the proposed sequential DEM-FEM framework is general and can be extended to other polymers with appropriate material-specific calibrations. For powder spreading, layer performance is evaluated based on surface profile, density, and uniformity. A method for partitioning and quantifying the powder layer is proposed, improving prediction accuracy of the spreading process window. PEEK and PEEK/CF powders—characterized by poor flowability and significant spreading difficulty—are selected as representative subjects. Their reasonable spreading window is clarified, revealing that layer thickness has a greater impact than spreading speed. Notably, the optimal layer thickness for PEEK/CF is slightly smaller than that for PEEK, indicating that adding CF particles reduces the minimum layer thickness, mitigating the staircase effect and improving dimensional accuracy. When thickness requirements are met, spreading speed can be increased to enhance efficiency. The optimal layer thickness for PEEK is determined to be 0.13 mm, establishing a key input condition for sintering. For the sintering stage, constraint criteria for sufficient sintering are established, and a systematic method for determining the sintering process window is proposed. By analyzing the microscopic mechanism of sufficient sintering, the influence of laser power, scanning speed, and hatch spacing is reflected through melt pool dimensions and temperature, enabling more efficient determination of a reasonable sintering window. Using the FEM model with the optimal layer thickness as input, a reasonable sintering process window for pure PEEK is determined, and its reliability is verified by experiments. The optimized process window for pure PEEK yields a maximum relative density of 99.31% and a 13.1% enhancement in tensile strength, indicating its potential for manufacturing load-bearing aerospace components such as brackets, clamps, and housings. Practical application to specific components would require further validation.

Future research will focus on four aspects: (1) macroscopic damage and SEM fracture analyses to reveal the failure mechanisms of PEEK specimens fabricated within the optimal process window; (2) experimental validation of PEEK/CF composite mechanical properties to complement the current DEM simulations; (3) extension of the proposed DEM-FEM framework to other high-performance thermoplastics such as PEKK and PPS; and (4) upscaling the optimized parameters to industrial-scale components including aerospace brackets, clamps, and drone structural frames; (5) development of a more comprehensive coupling strategy between DEM and FEM, e.g., by mapping spatially varying powder bed properties (such as local porosity distributions) from DEM simulations to the FEM model as initial conditions, to enable a more detailed investigation of how localized powder-bed heterogeneities affect melt pool dynamics and final part quality.

## Figures and Tables

**Table 1:** Average particle size distribution range of the PEEK and CF powders.

Type	Particle Size/μm	
	PEEK	CF
D_10_	23.884	6.39
D_50_	46.702	13.433
D_90_	84.546	77.043

**Table 2:** Particle contact parameters of PEEK [24].

Interaction Type	Static Friction Coefficient	Restitution Coefficient	Rolling Friction Coefficient	Surface Energy (J/m^2^)
PEEK–PEEK	0.4689	0.2746	0.1305	0.0050
PEEK–CF	0.4726	0.2695	0.1562	0.001
CF–CF	0.3833	0.0841	0.01	0.001
PEEK–wall	0.1429	0.2730	0.01	-
CF–wall	0.1021	0.0847	0.01	-

**Table 3:** Thermal and mechanical material properties of PEEK.

Mechanical Properties		Thermal Properties	
Elastic modulus/GPa	3.6	Thermal Conductivity/W·(m·°C)^−1^	0.29
Poisson’s ratio	0.38	Specific Heat Capacity /J·(kg·°C)^−1^	2180
density/g·cm^−3^	1.3	Glass Transition Temperature /°C	213.3
Yield Stress/MPa	107	Natural Convection Coefficient /W·(m^2^·K)^−1^	15

**Table 4:** Settings of roller spreading process parameters.

Powder Spreading Process Parameters	Symbol	Value
Roller translational speed /m·s^−1^	u	0.1, 0.2, 0.3, 0.4, 0.5
Roller rotational speed /rad·s^−1^	ω	5
Powder layer thickness /mm	t	0.1, 0.11, 0.12, 0.13, 0.14, 0.15

**Table 5:** Constraint criteria for determining contour and internal parameters to ensure adequate sintering of the powder layer.

Contour Parameters	Internal Parameters
t < d < 2t; T_max_ ∊ SSR	d = 1.5t; w > h
HAZ_min_; γ = w/2	T_max_ ∊ SSR

**Table 6:** Melt pool dimensions and temperature information of PEEK under different laser powers and scanning speeds.

No.	*P*/W	*v*/mm·s^−1^	*E_p_*/J·mm^−2^	*w*/mm	∆m/mm	*d*/mm	*T_max_*/°C
1	8	4000	0.0033	0	-	0	379.5140
2	8	3000	0.0044	0.1515	0.1515	0.0313	394.9690
3	12	3000	0.0067	0.2620	0.1200	0.0800	427.3840
4	20	4000	0.0083	0.3636	0.1004	0.1125	449.7810
5	16	3000	0.0089	0.3939	0.0960	0.1250	456.9640
6	20	3000	0.0111	0.4242	0.0871	0.1500	488.0120
7	32	4000	0.0133	0.4848	0.0849	0.1781	520.1120
8	20	2000	0.0167	0.5152	0.0814	0.2149	563.8830
9	25	2000	0.0208	0.5711	0.0780	0.2405	618.0160
10	16	1000	0.0267	0.5573	0.0744	0.2800	695.3570

**Table 7:** Contour and internal sintering parameters of PEEK.

Contour Parameters	Internal Parameters
Energy density *E_P_*: 0.018 J/mm^2^	Energy density *E_P_*: 0.015 J/mm^2^
Beam Offset γ: 0.266 mm	Hatching space *h*: <0.48 mm

**Table 8:** Experimental results of density and relative density of PEEK specimens.

Parameter Point	Measured Density/g·cm^−3^			Measured Density/g·cm^−3^ (Mean ± SD)	Relative Density/%			Relative Density/% (Mean ± SD)
	Sample 1	Sample 2	Sample 3		Sample 1	Sample 2	Sample 3	
A	1.22	1.22	1.20	1.21 ± 0.01	93.62	94.15	92.62	93.46 ± 0.77
B	1.28	1.28	1.29	1.28 ± 0.01	98.77	98.15	99.31	98.74 ± 0.58
C	1.24	1.25	1.25	1.25 ± 0.01	95.69	96.00	96.46	96.05 ± 0.39

**Table 9:** Tensile strength test results of PEEK specimens.

Laser Power/W	Scanning Speed/mm·s^−1^	Tensile Strength/Mpa
19	1500	48.98 ± 6.18
19	2000	56.34 ± 2.59
19	2500	53.68 ± 5.29

## Data Availability

The original contributions presented in this study are included in the article. Further inquiries can be directed to the corresponding author.
